# Influence of Fiber Deviation on Strength of Thin Birch *(Betula pendula Roth.)* Veneers

**DOI:** 10.3390/ma13071484

**Published:** 2020-03-25

**Authors:** Maximilian Pramreiter, Sabine C. Bodner, Jozef Keckes, Alexander Stadlmann, Cedou Kumpenza, Ulrich Müller

**Affiliations:** 1 Institute of Wood Technology and Renewable Materials, Department of Material Science and Process Engineering, University of Natural Resources and Life Sciences Vienna, Austria (BOKU), Konrad Lorenz Strasse 24, 3430 Tulln a.d. Donau, Austria; alexander.stadlmann@boku.ac.at (A.S.); cedou.kumpenza@boku.ac.at (C.K.); ulrich.mueller@boku.ac.at (U.M.); 2 Department of Materials Science, University Leoben, Jahnstrasse 12, 8700 Leoben, Austria; sabine.bodner@unileoben.ac.at (S.C.B.); jozef.keckes@unileoben.ac.at (J.K.)

**Keywords:** fiber-load angle, high performance composites, micro fibril angle, non-destructive testing, tensile strength, wide-angle X-ray scattering

## Abstract

The currently pursued implementation of wood into novel high performance applications such as automotive parts require knowledge about the material behaviour including ultimate strength. Previous research has shown that fiber deviation seems to be the dominating factor influencing the strength of thin veneers. This study aims to further investigate and quantify the influence of fiber deviation in two dimension and different hierarchical levels on the tensile strength of thin birch veneers. The fiber deviation in- and out-of-plane as well as the micro fibril angle were assessed by means of wide-angle X-ray scattering. Tensile strength was determined in laboratory experiments. Results show a high variability for in-plane fiber deviation mainly constituted by knots and other growth influencing factors. Pearson correlations between strength and fiber deviation ranged from −0.594 up to −0.852. Best correlation (r = −0.852) was achieved for maximum in-plane fiber deviation directly followed by a combined angle of in- and out-of-plane fiber deviation (r = −0.846). Based on the results it was shown that fiber deviation in- and out-of-plane is the dominating factor influencing ultimate tensile strength of thin birch veneers. Further research in regard to non-destructive strength prediction is necessary.

## 1. Introduction

The successful implementation of wood into high performance fields of application such as the automotive industry requires knowledge about the mechanical behaviour of the material on a piece by piece basis [1]. This includes not only information about the elastic properties but also ultimate strength [2]. To gain information about the mechanical properties of wood on a piece by piece basis different non-destructive techniques have been established already [3,4]. Most of these techniques are based on measuring relevant properties of the wood without impairing the end use capabilities of the material. Different strength influencing factors such as density, moisture content and fiber deviation have been frequently described in the literature [5,6,7]. Namely fiber deviation is assumed to be one of the most important properties affecting ultimate strength of wood. Kollmann [5] described a decrease in strength of more than 50% when the fiber deviates approximately 15° from the load axis. Recent literature on strength prediction based on fiber deviation deal with solid wood [8,9,10], Engineered Wood Products (EWP) mainly Laminated Veneer Lumber (LVL) [11,12] and veneers [13,14,15]. Viguier et al. [11] used optical scanners to measure grain angle on the surface of beech veneers for LVL production in order to predict elastic properties of the LVL beam. They only measured the surface angle of the two-millimetre-thick veneers, assuming a constant angle over the whole thickness. They conclude that measuring grain angle proofs to be a valuable tool in order to predict mechanical properties of veneers and EWP. Nevertheless, they also state that further investigations in regard to reliability are needed. Cha and Pearson [12] investigated the influence of cracks in the middle layer on the overall stress distribution using Finite-Element-Modelling (FEM) to predict stress distributions within a LVL beam. They concluded that different grain angles strongly influence the stress distribution within the material. Lang et al. [14] examined the influence of grain angle on the elastic properties of different hardwood veneers. Focusing mainly on elastic properties they demonstrated good predictability of modulus of elasticity based on the variation of grain angles. However, they did not assess the possibility to also predict strength based on different fiber deviations. Previous research on thin birch *(Betula pendula Roth.)* veneers [15] has shown that the fiber deviation seems to have a much stronger influence on the ultimate strength than the dynamic modulus of elasticity or density. Therefore, measuring the fiber angle promises to enable a non-destructive and precise sorting on a piece by piece basis.

To meet design requirements of the automotive industry most of the components in a vehicle have a three dimensional spherical structure [16]. To be competitive with fossil-based counterparts made of glass or carbon-fiber wood-based components will predominantly made of thin multilayer composites [17,18]. Therefore, the layer thickness of the wood-based composites is in the range of millimetres rather than centimetres in order to be processible [19].

The present study aims to further investigate and quantify the influence of fiber deviation on tensile strength of thin birch *(Betula pendula Roth.)* veneers with a thickness of one millimetre or less. The fiber angles are determined by means of wide-angle X-ray scattering (WAXS), which is used to evaluate the fiber deviation in two dimensions and at different hierarchical levels. The two main hypotheses the study aims to answer are:

- Wide-angle X-ray scattering delivers reliable fiber deviations in two dimensions as well as at two hierarchical levels.
- A sound correlation between fiber deviation and ultimate tensile strength of the veneer can be achieved.

## 2. Materials and Methods

### 2.1. Sample Preperation

**Figure 1:**
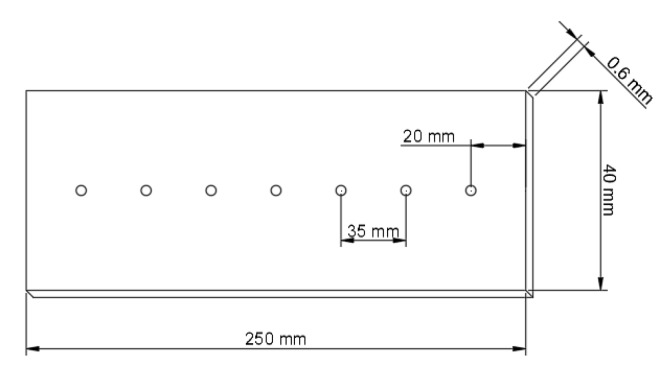
Sample geometry and measuring spots for WAXS.

A total of 20 samples including two dummy samples, with a thickness of approximately 0.6 mm ± 0.05 mm, a width of 40 mm ± 1 mm and a length of 250 mm ± 1 mm, were cut out of unsorted Finnish birch *(Betula pendula*) (sourced from Koskisen, Järvelä, Finland) veneers with original dimensions of 1300 mm × 1300 mm using a circular saw. The batch included a random distribution of the different quality grades as described by Koskisen [20]. The sample geometry as well as the measuring spots for the WAXS tests is further depicted in Figure 1. The sample size was mainly chosen due to the high time demand of the wide-angle X-ray scattering technique. Prior to testing, the specimens were stored under standard climate conditions at 20 °C ± 2 °C and 65% ± 5% relative humidity (RH) in accordance to standard ISO 554 [21] until an equilibrium moisture content of approximately 12% was reached. The thickness of each individual sample was determined within an accuracy of ± 0.01 mm before testing on using a digital caliper (Series 500, Mitutoyo, Neuss, Germany).

### 2.2. Sample Properties

The density (*ρ*) of the samples was calculated according to the standard DIN 52182 [22] using their mass (*m*) and volume (*V*) obtained by their dimensions.

The tensile strength (*σ*_t_) of the 18 samples was determined according to standard DIN 789 [23] by using a universal testing machine (Z100, Zwick/Roell, Ulm, Germany) with a cell capacity of 100 kN and a resolution of 0.06 N. Mounting the samples in the setup resulted in a free testing length of 210 mm. The specimens were pre-loaded with 100 N to compensate for possible distortions due to conditioning. A testing speed of 2 mm/min was chosen to reach a testing time of 300 s ± 120 s. The test was stopped after a 30% force reduction was reached. The strength was calculated according to standard DIN EN 789 [23], as shown in Equation (1),

(1) $$\sigma_{t\;}=\frac{F_{\max}}{A}$$

where σ_t_ is the tensile strength (MPa); *F*_max_ is the peak force (N); and *A* is the cross-sectional area of the sample (mm²).

### 2.3. Wide-Angle X-Ray Scattering (WAXS)

**Figure 2:**
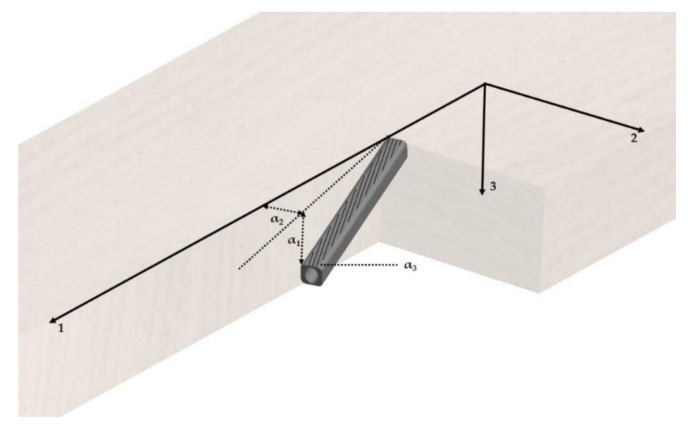
Possible deviations of the fiber within the wood and deviations of the fibrils within the fibers. α1 describes the angle between axis 1 (usually the longitudinal direction) and axis 3 (either tangential or radial direction). α2 describes the angle between axis 1 and axis 2 (either radial of tangential direction). α3 illustrates the deviation of the fibril from the fiber axis in the cell wall also known as micro fibril angle (mfa). [own depiction].

The possible fiber deviations in- (α2) and out-of-plane (α1) as well as micro fibril angle (mfa) within the axially cut wood sections with a thickness of 0.6 mm ± 0.05 mm are depicted in Figure 2. To characterize the possible fiber deviations wide-angle X-ray scattering measurements were performed at the high energy materials science (HEMS) beamline P07B of PETRA III at Deutsches Elektronen-Synchrotron (DESY) in Hamburg. The installation is operated by Helmholtz Zentrum Geesthacht. The experiments were performed in transmission diffraction geometry using a monochromatic beam with an energy of 87.1 keV and a beam cross-section of ~(500 × 500) µm. The diffraction data were collected using a Perkin Elmer two-dimensional (2D) flat panel detector of 2048 × 2048 pixels with a pixel pitch of ~200μm positioned at the distance of ∼1.3 m form the samples. The measured sample positions within the veneers are depicted in Figure 1.

The diffraction data were used to evaluate average magnitudes of fiber deviation (α1, α2 and mfa) in the irradiated sample gauge volumes (with the precision of ~±1 degree) from the azimuthal positions of cellulose 200 reflections appearing along cellulose 200 Debey-Scherrer rings, as described by Lichtenegger et al. [24]. To further investigate the interactions between fiber deviation in- and out-of-plane with the strength of the veneers a combined angle of α1 and α2 was calculated according to Equation (2) [25],

(2) $$\beta =cos^{-1}\times \left(\cos\frac{\alpha 1}{180\pi }\times cos\frac{\alpha 2}{180\pi }\right)\times \frac{180}{\pi }$$

where β is the combined angle (°); α1 is the deviation out-of-plane (°) and α2 is the deviation in-plane (°).

Recorded data were processed using Excel 2016 (Microsoft, Redmond, Washington, DC, USA). Mean, minimum and maximum values where determined applying the built-in functions. The coefficient of variance (COV) was calculated using the built-in function for standard deviation and the respective mean value. Furthermore, the correlation between the fiber deviation and the tensile strength of the veneers was determined using the built-in Pearson function.

## 3. Results and Discussion

### 3.1. Sample Properties

Table 1 summarizes the measured parameters for the tensile strength (*σ*_t_), the density (*ρ*), the micro fibril angle (mfa), the out-of-plane fiber deviation between axis 1 and axis 3 (α1), the in-plane fiber deviation between axis 1 and axis 2 (α2) and the combined angle according to Equation (2) between α1 and α2 (β).

The tensile strength of the veneers was between 36.76 MPa and 160.28 MPa with an average of 95.03 MPa and a coefficient of variance (COV) of 37%. The high variability in strength can be explained due to the unsorted sample batch including almost defect free veneers as well as knotty or sloppy grained samples. The results for tensile strength are also below typical results in the literature, reporting an average strength of around 140 MPa [26] or even as high as 270 MPa [27]. This deviation can again be explained with the unsorted sample batch as usual literature values are established on defect free, fine grained samples with little to no fiber deviation. Additionally, the measured density of 605 kg/m³ is also below typical literature values reporting an average density at 12–15% moisture content of around 650 kg/m³ [26] up to 830 kg/m³ [27]. This in turn could also explain the lower strength as density greatly influences the strength of wood in general [7].

The micro fibril angle ranged from 7.54° to 12.89° with an average of 9.61°. These results are similar to literature values stating an average mfa of 10°–12° for mature birch wood [28]. The fiber deviation out-of-plane (1.61°) was slightly lower than the fiber deviation in-plane (2.83°). In general, the fiber deviation showed very high variability with a COV of 39% for α1 and 63% for α2 respectively. Also the variability of the in- and out-of-plane angles could be described due to defects such as knots, trunk curvature or crookedness, tapering and buttress roots. The fiber deviation around knots is a very complex but common occurrence within wood. Mainly effected by the growth of the tree [29,30] fibers in the vicinity of knots deviate heavily from the dominating fiber direction in- as well as out-of-plane [31]. Trunk curvature or crookedness either occurs due to genetic disposition or as a result of external forces [32]. In both cases the change in growth direction of the trunk leads to change in fiber orientation. Mainly driven by competition during growth [32], highly tapered trunks lead to a higher fiber deviation within the cut veneer [19]. Buttress roots mainly act as a support against mechanical stresses due to wind in tropical trees [33] but can also be found in European solid woods [34]. Fiber direction and buttress formation will change depending on the direction of the mechanical stress in order to “pull” the stem into an upright position [35].

### 3.2. Fiber Deviation

**Figure 3:**
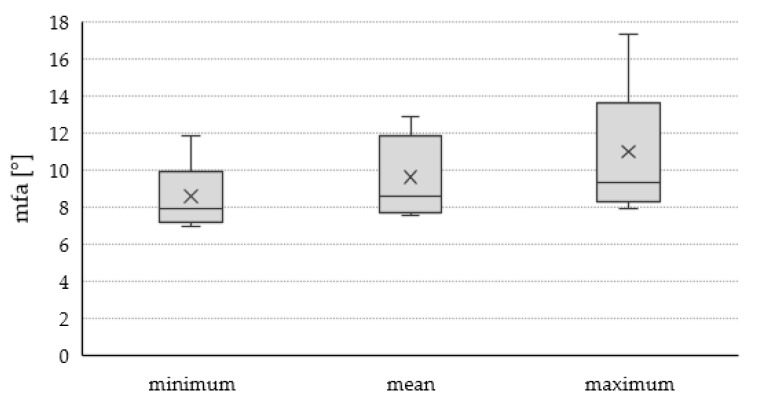
Minimum, mean, and maximum micro fibril angle of n = 18 samples. The mean value is an average of seven measurements within one sample. Minimum and maximum are lowest and highest recorded value of one sample respectively.

The variability of the mfa within the samples is relatively low, as can be seen in Figure 3. However, there is some degree of variability. The reason for this can be manifold. The provenance of the trees could influence the mfa within the tree and therefore influence the resulting mfa of the different veneers. However, research is scarce [36,37] and only low correlation between provenance and mfa was found for hardwoods. Another possible explanation could be the position of the veneers within the original stem [38,39]. According to Barnett and Bonham [39], the mfa decreases from pith to bark. Therefore, a veneer originating close to the pith would exhibit a higher mfa than ones closer to the bark. A similar relationship exists in regard to the originating height of the veneer within the tree. For hardwoods, a decreases in mfa is observed along the height of a log, which may explain higher mfa values for veneers gained closer to the log bases [37]. Additionally, a certain variability could exist due to differences in early- and latewood content and the presents of reaction wood. According to Fang et al. [40] mfa in latewood is 1–5° lower than in earlywood. Furthermore, reaction wood in hardwoods leads to a “straightening” of the mfa in the so-called gelatinous layer [39]. Both factors would lead to a lower bulk mfa in veneers with either higher latewood content or reaction wood respectively.

**Figure 4:**
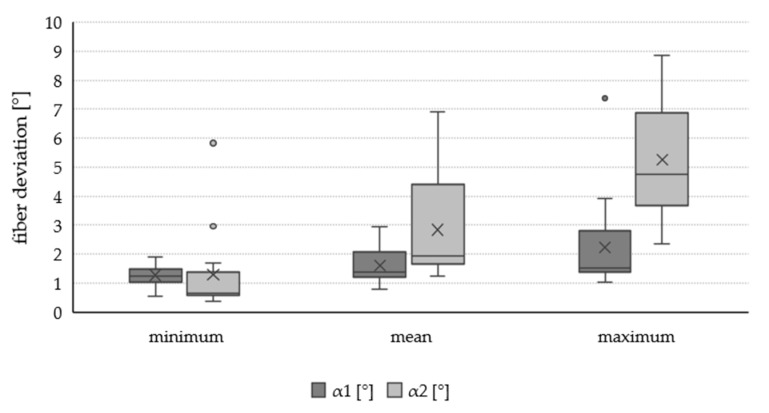
Minimum, mean and maximum fiber deviation in- and out-of-plane of n = 18 samples. The mean value is an average of seven measurements within one sample. Minimum and maximum are lowest and highest recorded value of one sample respectively.

**Figure 5:**
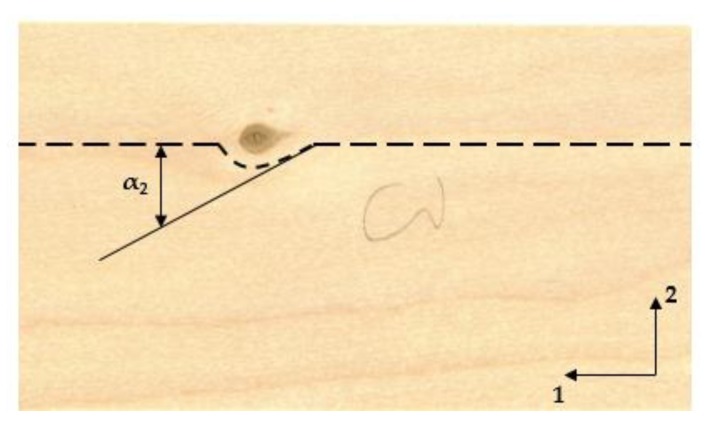
In-plane fiber deviation in the vicinity of a knot. Axis 1 represents the longitudinal direction of the veneer and axis 2 either the tangential or the radial direction respectively.

Compared to the mfa the variability of the fiber deviation is significantly higher, especially for the in-plane deviation α2. Figure 4 compares the fiber deviation in- and out-of-plane of all 18 samples. As mentioned previously, fiber deviation in the vicinity of knots is complex and usually vastly different from the rest of the wood material [29,41]. The deflection α2 mainly occurs when fibers are deviated at the vicinity of knots in the longitudinal direction of the veneer. This explains the high variability of α2 to a certain extend. The deviation is further depicted in Figure 5.

Based on findings in the literature [31,42], it was expected that the deviation out-of-plane α1 also exhibits high variability due to knots. However, the observed variability (see Figure 4) is rather low compared to the in-plane deviation α2. As reported by Foley [42] (p. 463), α1 normalizes faster compared to α2 which influences a much bigger area around the knot. Therefore, the measuring area of the out-of-plane deviation is much more sensitive. Placing the measuring area too far away from the knot could lead to an underestimation of α1 and furthermore report a too low bulk α1 for the whole sample. According to Stahl [43] the heaviest affected area is approximately on knot radius around the visual border of the knot.

### 3.3. Fiber Deviation and Strength

The correlation between the different angles investigated in this study and the respective strength of the sample is depicted in Table 2. In general, all angles correlate negatively with strength. This was expected as strength parallel to the fiber is approximately ten times higher than perpendicular [5,7]. Therefore, any off-axis loading leads to a decrease in strength. Best correlation was achieved with mean β (r = −0.846) and maximum α2 (r = −0.852). Correlation with mean α2 (r = −0.806) as well as maximum β (r = −0.818) was also high but considerably lower. Other correlations including all minimum angles were still significant but exceedingly below 0.800.

**Figure 6:**
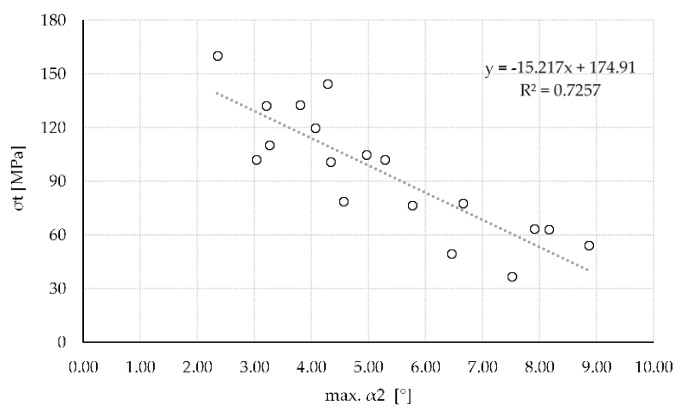
Influence of max. α2 on the tensile strength of n = 18 samples (r = −0.852).

Figure 6 further depicts the influence of maximum α2 on the strength of the veneers. The influence of in-plane fiber deviation on strength seems to follow a rather linear relationship. This contradicts literature [5,7] stating a high decrease of strength between 3° and 30° and a more constant level onwards. Yet, no extreme values (0° or 90°) for fiber deviation were observed in the tested sample batch. Therefore, neither the top or the bottom values for strength were established. The full curve could still reassemble the behaviour mentioned by literature.

**Figure 7:**
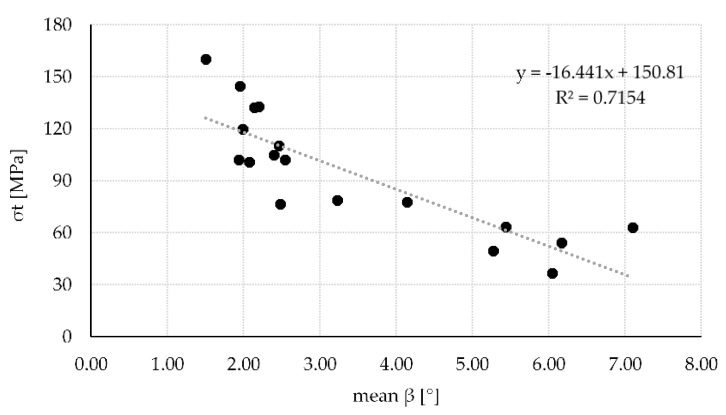
Influence of mean β on the tensile strength of n = 18 samples (r = −0.846).

**Figure A1:**
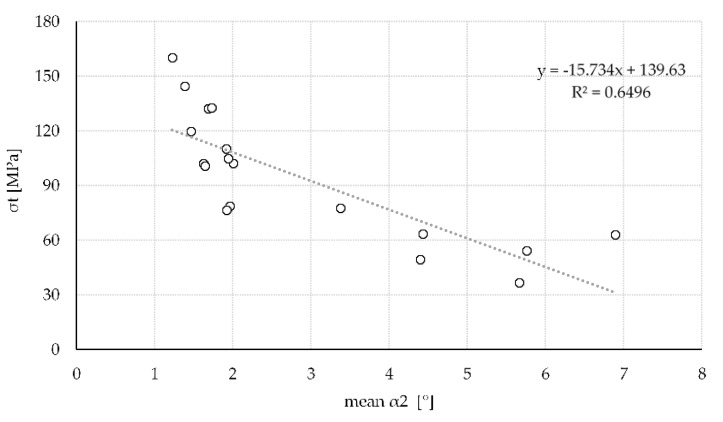
Influence of mean α2 on the tensile strength of n = 18 samples (r = −0.806).

**Figure A2:**
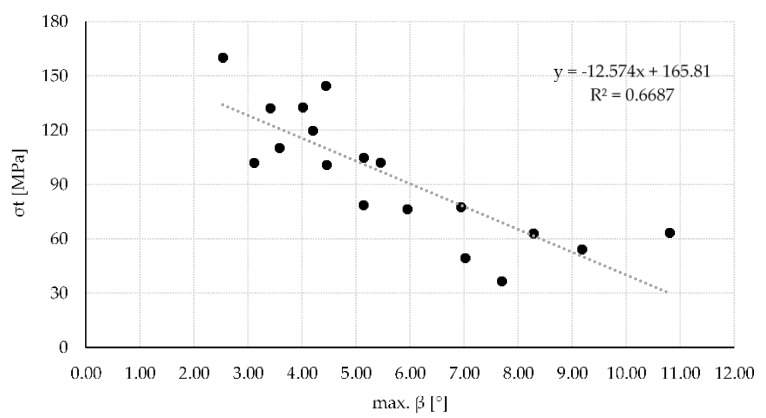
Influence of maximum β on the tensile strength of n = 18 samples (r = −0.818).

Figure 7 illustrates the influence of mean β on the strength of the veneers. Compared to α2 the influence of β on strength appears to be non-linear. There is a bigger decrease in strength between 2° and 4° compared to 4° onwards. Part of that is in line with literature mentioned earlier [5,7]. However, the gradient seems to be higher than ones stated in the literature as strength decreases more than 50% when the fiber deviates only 2° from the longitudinal axis. Kollmann [5] (p. 669) stated a 50% decrease in strength over an 15° change in fiber deviation. A similar relationship was also stated by Dinwoodie [44]. Further graphs showing the influence of mean α2 (see Figure A1) as well as maximum β (see Figure A2) can be found in Appendix A.

As mentioned in the previous section in- as well as out-of-plane fiber deviation occurs pre-dominantly in the vicinity of knots [45]. Other reasons for the fiber deviation could be spiral grain, reaction wood, accumulation of defects [46] and climate driven changes in growth [33]. No matter why the fiber deviation is generated the influence on strength is present either way. Furthermore, it seems that the in-plane deviation is the dominating factor influencing the tensile strength of thin veneers. Yet, there is also an interaction between in- and out-of-plane deviation in regard to strength prediction. A model predicting strength should therefore include both angles. Considering already established failure criteria after Hakinson [47], Kollmann [48], Hoffmann [49], or Tsail-Hill [50] only one angle is applicable. Combining the angles with Equation (2) could improve the overall predictability of strength based on these failure criteria. Further research investigating already established failure criteria and their applicability on thin wooden veneers is necessary.

## 4. Conclusions

The aim of the present paper was to investigate the influence of fiber deviation in- and out-of-plane as well as the micro fibril angle on the tensile strength of thin birch *(Betula pendula Roth.)* veneers. The fiber deviation was determined by means of wide-angle X-ray scattering method (WAXS). Based on the results different conclusions can be drawn.

The WAXS technique delivers reliable fiber deviations on two different hierarchical levels (fiber and cell wall level) as well as two dimensions (in- and out-of-plane). On the downside is a high time demand for the measuring setup and the measurement itself. An industrial implementation would need further improvements in regard to measurement duration and sensitivity of the setup.

It was proven that the fiber deviation is the dominating factor regarding ultimate tensile strength of thin veneers. A model predicting strength should therefore be mainly based on the measured fiber deviation.

The influence of fiber deviation in- as well as out-of-plane on tensile strength is in line with literature. An increase in fiber deviation in- or out-of-plane leads to a significant decrease in ultimate strength. The initial decrease in strength, however, is significantly higher than in the literature.

The influence of micro fibril angle on tensile strength is significant and in line with literature. However, the influence of fiber deviation in- and out-of-plane shows higher correlation further supporting their capabilities to predict strength of thin birch veneers.

## Figures and Tables

**Table 1:** Overview of the determined material properties of n = 18 samples. The different angles (mfa, α1 and α2) are furthermore an average of seven measuring points within the veneer.

Statistical Indicator	σ_t_ [MPa]	ρ [kg/m³]	mfa [°]	α1 [°]	α2 [°]	β [°]
n	18	18	18	18	18	18
mean	**95.03**	605	**9.61**	**1.61**	**2.83**	**3.39**
COV	37%	13%	23%	39%	63%	53%
min.	36.76	469	7.54	0.79	1.22	1.50
max.	160.28	684	12.89	2.94	6.89	7.10

**Table 2:** Pearson correlation of the relevant properties’ density, minimum, mean, and maximum α1, α2 and mfa with the ultimate tensile strength of n = 18 samples.

Property	Min. Angle	Mean Angle	Max. Angle
σ_t_ [MPa]	1	1	1
ρ [kg/m³]	0.551	0.551	0.551
mfa [°]	−0.726	−0.805	−0.783
α1 [°]	−0.663	−0.762	−0.594
α2 [°]	−0.626	−0.806	**−0.852**
β [°]	−0.735	**−0.846**	−0.818
